# Supplementary material for: Predicting sediment and nutrient concentrations from high-frequency water-quality data
Source: PLoS One. 2019 Aug 30;14(8):e0215503. doi: 10.1371/journal.pone.0215503 (PMC6716630; doi:10.1371/journal.pone.0215503)
Supplement: S1 Table — The best NOx model for each site based on the cvRMSE (i.e. lowest value per site), following backwards stepwise selection of the covariates in each model using the Akaike Information Criterion. (DOCX) [file pone.0215503.s002.docx]

Supporting Information

For the main article, “Predicting sediment and nutrient concentrations from high-frequency water-quality data” by Catherine Leigh, Sevvandi Kandanaarachchi, James M. McGree, Rob J. Hyndman, Omar Alsibai1, Kerrie Mengersen and Erin E. Peterson, published by Plos One.

This document contains S1 Table.

**S1 Table. Site-based NOx models.** The best NOx model for each site based on the *cvRMSE* (i.e. lowest value per site), following backwards stepwise selection of the covariates in each model using the Akaike Information Criterion.

| **Site** | **Level category** | **Covariates in the model (log_10_-transformed)** | ***cvRMSE***  ***(log_10_-scale)*** |
| --- | --- | --- | --- |
| **MR** | <Q1 | Conductivity + Turbidity + Conductivity*Turbidity | 0.0355 |
|  | <Q2 | Conductivity | 0.0322 |
|  | <Q3 | Conductivity | 0.0332 |
| **PR** | <Q1 | Conductivity + Level + Turbidity + Conductivity*Level + Conductivity*Turbidity + Turbidity*Level + Conductivity*Level*Turbidity | 0.1239 |
|  | <Q2 | Level + Turbidity + Level*Turbidity | 0.0884 |
|  | <Q3 | Conductivity + Level + Conductivity*Level | 0.1051 |
| **SC** | <Q1 | Conductivity + Level + Turbidity + Conductivity*Level + Conductivity*Turbidity | 0.1730 |
|  | <Q2 | Conductivity + Level + Conductivity*Level | 0.1541 |
|  | <Q3 | Conductivity + Level + Turbidity + Conductivity*Level + Conductivity*Turbidity | 0.1656 |
