# Supplementary material for: Predicting sediment and nutrient concentrations from high-frequency water-quality data
Source: PLoS One. 2019 Aug 30;14(8):e0215503. doi: 10.1371/journal.pone.0215503 (PMC6716630; doi:10.1371/journal.pone.0215503)
Supplement: S1 Fig — The relationship between laboratory-determined turbidity (NTU) and total suspended solids (TSS, mg/L), log10-transformed, at Mulgrave River (MR; left plot), Pioneer River (PR, middle plot) and Sandy Creek (SC, right plot), with turbidity values < 15 NTU represented by closed triangles and those ≥ 15 NTU by closed circles, with each set in the freshwater sites PR and SC enclosed by ellipses. (DOCX) [file pone.0215503.s003.docx]

Supporting Information

For the main article, “Predicting sediment and nutrient concentrations from high-frequency water-quality data” by Catherine Leigh, Sevvandi Kandanaarachchi, James M. McGree, Rob J. Hyndman, Omar Alsibai1, Kerrie Mengersen and Erin E. Peterson, published by Plos One.

This document contains S1 Fig.

**
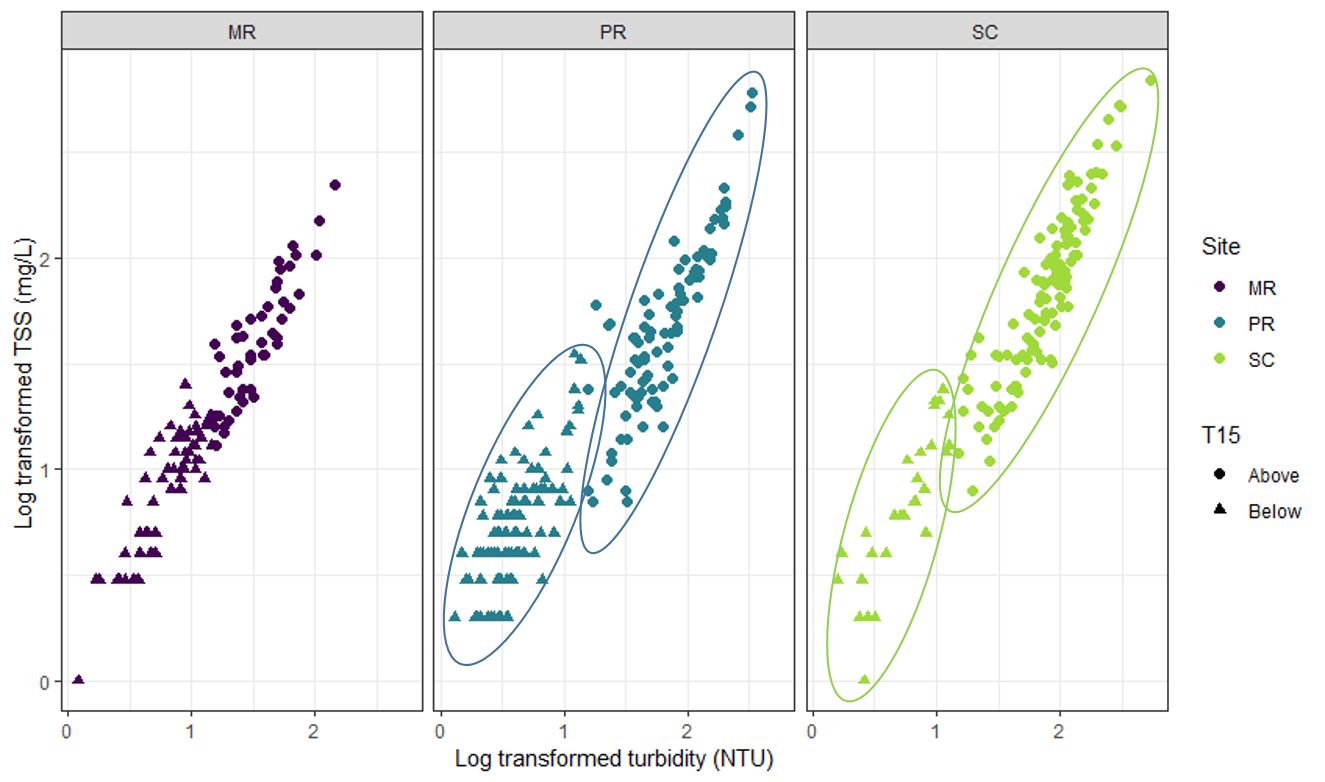
 S1 Fig. Turbidity and TSS.** The relationship between laboratory-determined turbidity (NTU) and total suspended solids (TSS, mg/L), log_10_-transformed, at Mulgrave River (MR; left plot), Pioneer River (PR, middle plot) and Sandy Creek (SC, right plot), with turbidity values < 15 NTU represented by closed triangles and those ≥ 15 NTU by closed circles, with each set in the freshwater sites PR and SC enclosed by ellipses.
