# Supplementary material for: Predicting sediment and nutrient concentrations from high-frequency water-quality data
Source: PLoS One. 2019 Aug 30;14(8):e0215503. doi: 10.1371/journal.pone.0215503 (PMC6716630; doi:10.1371/journal.pone.0215503)
Supplement: S4 Fig — The relationship between river level (m) recorded on-site at the time of water sample collection and oxidized nitrogen (NOx, mg/L), log10-transformed, at Mulgrave River (MR; left plot), Pioneer River (PR, middle plot) and Sandy Creek (SC, right plot). (DOCX) [file pone.0215503.s006.docx]

Supporting Information

For the main article, “Predicting sediment and nutrient concentrations from high-frequency water-quality data” by Catherine Leigh, Sevvandi Kandanaarachchi, James M. McGree, Rob J. Hyndman, Omar Alsibai1, Kerrie Mengersen and Erin E. Peterson, published by Plos One.

This document contains S4 Fig.

**
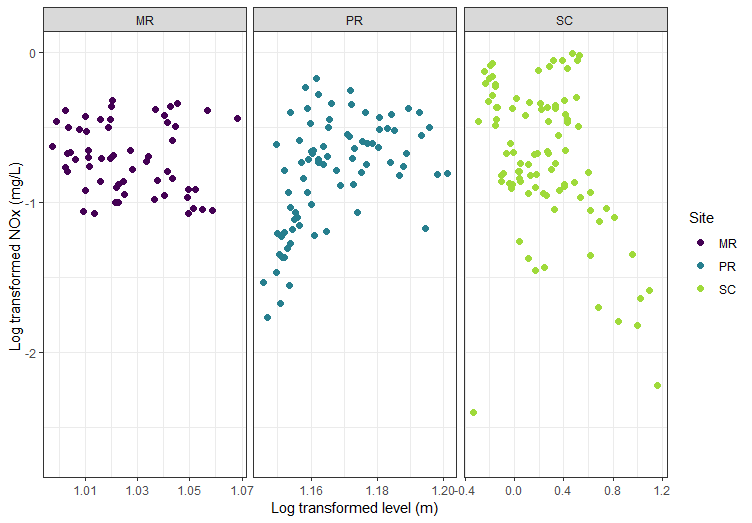
 S4 Fig. River level and NOx.** The relationship between river level (m) recorded on-site at the time of water sample collection and oxidized nitrogen (NOx, mg/L), log_10_-transformed, at Mulgrave River (MR; left plot), Pioneer River (PR, middle plot) and Sandy Creek (SC, right plot).
