# Supplementary material for: Predicting sediment and nutrient concentrations from high-frequency water-quality data
Source: PLoS One. 2019 Aug 30;14(8):e0215503. doi: 10.1371/journal.pone.0215503 (PMC6716630; doi:10.1371/journal.pone.0215503)
Supplement: S6 Fig — Diagnostic plots for the final total suspended solids (TSS, mg/L, log10-transformed) model. Upper row: fitted values vs residuals. Middle row: boxplots of residuals. Lower row: QQ-plot. Left column: by site (MR, Mulgrave River; PR, Pioneer River; SC, Sandy Creek). Right column: by T15 (Above: turbidity ≥ 15 NTU; Below: turbidity < 15 NTU). (DOCX) [file pone.0215503.s008.docx]

Supporting Information

For the main article, “Predicting sediment and nutrient concentrations from high-frequency water-quality data” by Catherine Leigh, Sevvandi Kandanaarachchi, James M. McGree, Rob J. Hyndman, Omar Alsibai1, Kerrie Mengersen and Erin E. Peterson, published by Plos One.

This document contains S6 Fig.

| 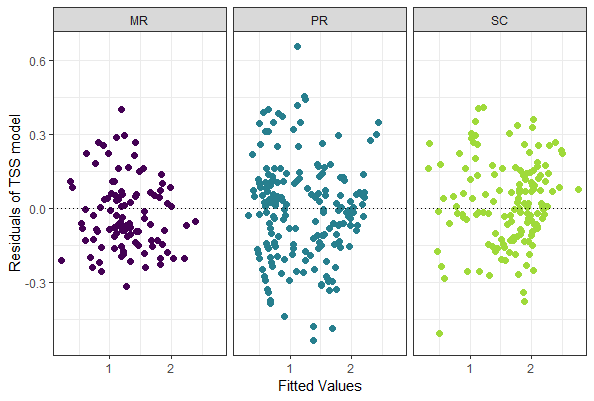 | 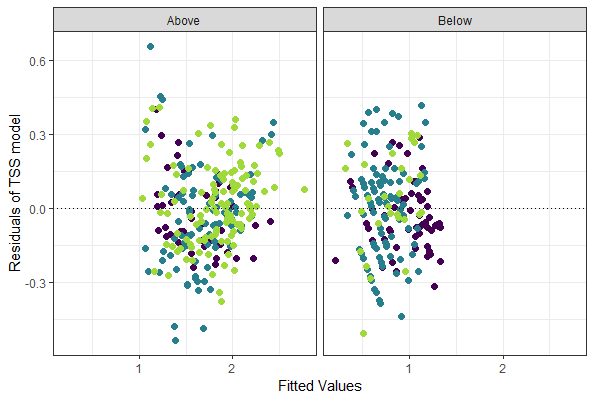 |
| --- | --- |
| 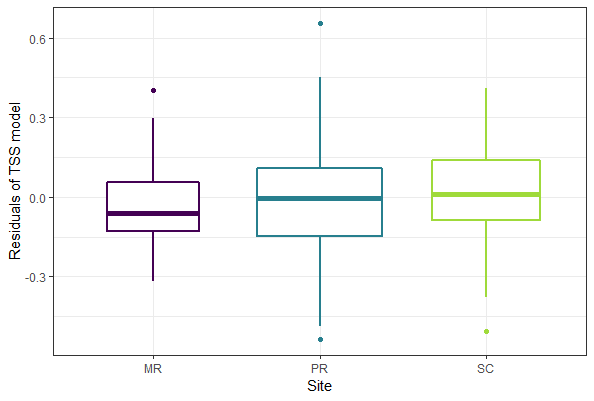 | 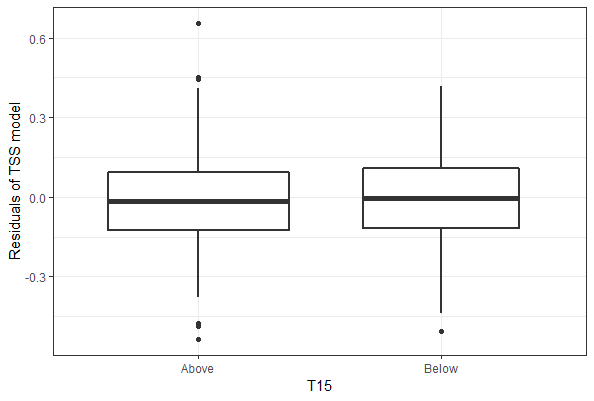 |
| 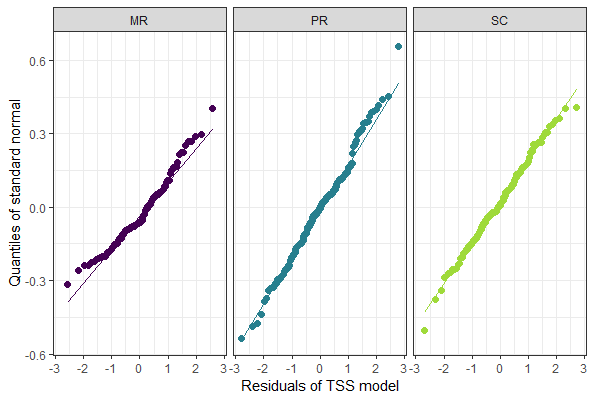 | 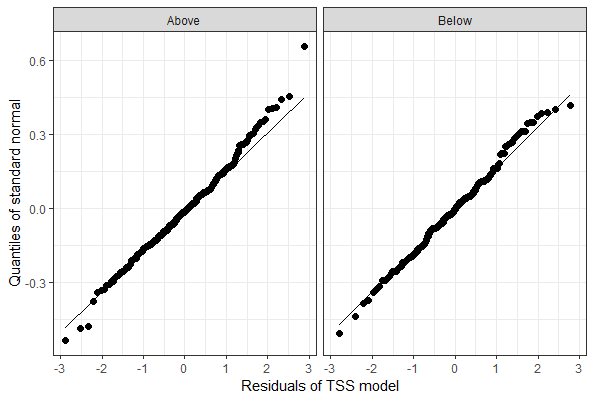 |

**S6 Fig. TSS-model diagnostic plots.** Diagnostic plots for the final total suspended solids (TSS, mg/L, log_10_-transformed) model. Upper row: fitted values vs residuals. Middle row: boxplots of residuals. Lower row: QQ-plot. Left column: by site (MR, Mulgrave River; PR, Pioneer River; SC, Sandy Creek). Right column: by T15 (Above: turbidity ≥ 15 NTU; Below: turbidity < 15 NTU).
