# Supplementary material for: Predicting sediment and nutrient concentrations from high-frequency water-quality data
Source: PLoS One. 2019 Aug 30;14(8):e0215503. doi: 10.1371/journal.pone.0215503 (PMC6716630; doi:10.1371/journal.pone.0215503)
Supplement: S7 Fig — Residuals from the final oxidized nitrogen (NOx, mg/L, log10-transformed) models plotted in chronological order of the observations at Mulgrave River (MR; upper plot), Pioneer River (PR, middle plot) and Sandy Creek (SC, lower plot). (DOCX) [file pone.0215503.s009.docx]

Supporting Information

For the main article, “Predicting sediment and nutrient concentrations from high-frequency water-quality data” by Catherine Leigh, Sevvandi Kandanaarachchi, James M. McGree, Rob J. Hyndman, Omar Alsibai1, Kerrie Mengersen and Erin E. Peterson, published by Plos One.

This document contains S7 Fig.


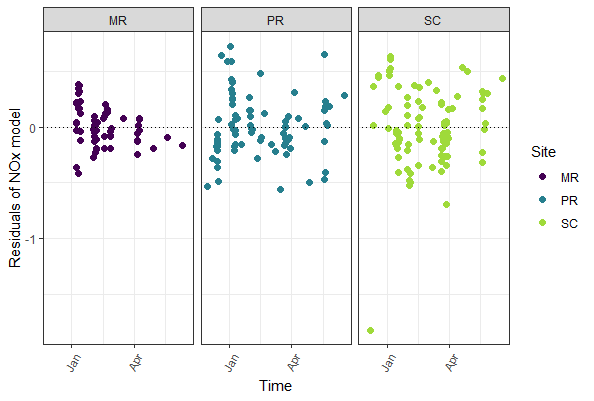


**S7 Fig. NOx-model residuals.** Residuals from the final oxidized nitrogen (NOx, mg/L, log_10_-transformed) models plotted in chronological order of the observations at Mulgrave River (MR; upper plot), Pioneer River (PR, middle plot) and Sandy Creek (SC, lower plot).
