# Supplementary material for: Predicting sediment and nutrient concentrations from high-frequency water-quality data
Source: PLoS One. 2019 Aug 30;14(8):e0215503. doi: 10.1371/journal.pone.0215503 (PMC6716630; doi:10.1371/journal.pone.0215503)
Supplement: S8 Fig — Diagnostic plots for the final oxidized nitrogen (NOx, mg/L, log10-transformed) model for each site (Mulgrave River, MR; purple points; Pioneer River, PR; blue points; Sandy Creek, SC; light green points). Upper row: fitted values versus residuals. Middle row: boxplots of residuals. Lower row: QQ-plots. (DOCX) [file pone.0215503.s010.docx]

Supporting Information

For the main article, “Predicting sediment and nutrient concentrations from high-frequency water-quality data” by Catherine Leigh, Sevvandi Kandanaarachchi, James M. McGree, Rob J. Hyndman, Omar Alsibai1, Kerrie Mengersen and Erin E. Peterson, published by Plos One.

This document contains S8 Fig.

| 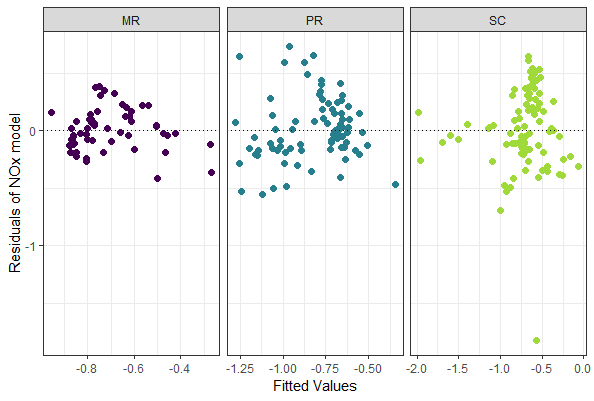 |
| --- |
| 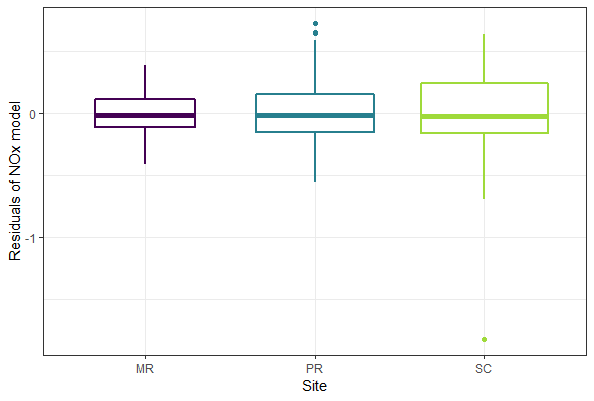 |
| 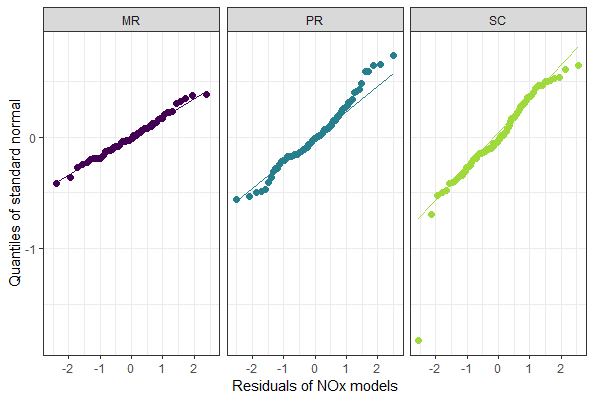 |

**S8 Fig.** **NOx-model diagnostic plots.** Diagnostic plots for the final oxidized nitrogen (NOx, mg/L, log_10_-transformed) model for each site (Mulgrave River, MR; purple points; Pioneer River, PR; blue points; Sandy Creek, SC; light green points). Upper row: fitted values versus residuals. Middle row: boxplots of residuals. Lower row: QQ-plots.
